# A Large‐Scale Retrospective Study of Serum Des‐Gamma‐Carboxy Prothrombin as a Diagnostic Marker of HCC: Effect of Liver Function on Specificity

**DOI:** 10.1002/jcla.70025

**Published:** 2025-04-14

**Authors:** Hongying Bu, Weijia Luo, Wenli Tao, Chen Dong, Meifang Wang, Xu Ye, Xi Zeng, Boqing Wang, Chang Liu, Qi Yu, Deliang Cao, Hongyu Deng, Yuemin Nan

**Affiliations:** ^1^ School of Public Health, Hengyang Medical School University of South China Hengyang China; ^2^ Hunan Engineering Research Center for Early Diagnosis and Treatment of Liver Cancer, Hunan Province Key Laboratory of Tumor Cellular and Molecular Pathology, Cancer Research Institute, Hengyang Medical School University of South China Hengyang Hunan China; ^3^ Provincial Key Laboratory of Study on Mechanism of Hepatic Fibrosis in Chronic Liver Disease, Department of Traditional and Western Medical Hepatology Hebei Medical University Third Hospital Shijiazhuang China; ^4^ Science and Technology Innovation Center Hunan University of Chinese Medicine Changsha China; ^5^ The Affiliated Cancer Hospital of Xiangya School of Medicine Central South University/Hunan Cancer Hospital Changsha China; ^6^ Department of Hepatopancreatobiliary Surgery The Affiliated Tumor Hospital of Xinjiang Medical University Xinjiang China; ^7^ Engineering and Research Center for Integrated New Energy Photovoltaics & Energy Storage Systems of Hunan Province and School of Electrical Engineering University of South China Hengyang China

**Keywords:** AFP, DCP, HCC, retrospective study, serum biomarker

## Abstract

**Background:**

This retrospective multicenter study is aimed at evaluating the diagnostic accuracy and influence factors of serum des‐gamma‐carboxy prothrombin (DCP) as a diagnostic biomarker of hepatocellular carcinoma (HCC).

**Methods:**

Clinical data were collected from 4555 subjects with DCP tests, composed of primary liver cancer (PLC), metastatic liver cancer (MLC), chronic hepatitis (CH), liver cirrhosis (LC), benign liver diseases (BLD), biliary tract diseases (BTD), non‐liver cancers (NLC), and non‐liver benign diseases (NLBD). The clinical data collected included medical history, treatment records, various serum tests, and imaging examination.

**Results:**

Serum DCP was measured with Abbott agents in each center. In HCC, serum DCP concentration was at 9086.00 ± 366.10 mAU/mL, higher than that in other diseases (*p* < 0.05). At 40.00 mAU/mL recommended by instruction, positive rates of serum DCP were at 85.11% in HCC, 30.12% in intrahepatic cholangiocellular carcinoma (ICC), 31.65% in MLC, 13.95% in BLD, 18.14% in CH, 27.87% in LC, 15.75% in BTD, 35.29% in NLC, and 20.00% in NLBD. In this study, the diagnostic specificity of serum DCP in HCC was affected by liver function. In HCC, serum AFP concentrations also increased compared to non‐HCC diseases (*p* < 0.05), but specificity varied with agents from different providers. Serum DCP decreased after the surgical removal of HCC, but remained elusive in systemic treatment.

**Conclusion:**

Serum DCP may serve as an optimal biomarker for the diagnosis of HCC, but its accuracy appears influenced by liver function; attention needs to be paid to the liver function of patients for false positivity.

## Graphical abstract

Serum DCP may serve as an optimal biomarker for the diagnosis of HCC, but its accuracy appears to be influenced by liver function. The serum DCP may also serve as a biomarker for liver resection and interventional therapy of HCC, but is not promising for systemic treatment.

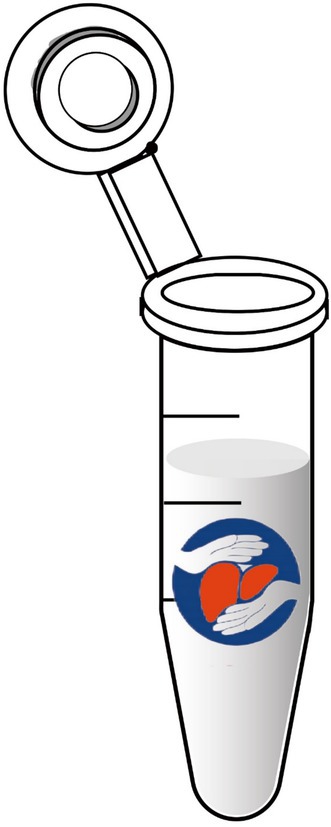

## 1 Introduction

Primary liver cancer (PLC) arises from hepatocytes or intrahepatic cholangiocytes, and hepatocellular carcinoma (HCC) derived from hepatocytes is the dominant type, accounting for 85%–90% of PLC [1]. Global statistics in 2020 indicated that 905,677 new cases of HCC were diagnosed, ranked as the second cancer‐related death [2]. Up to 75% of HCC occurs in Asia, and China has nearly 50% of the total [3]. Several studies have indicated that the median survival for HCC is less than 1 year, and the average rate of survival for 5 years is less than 15% [4, 5]. The World Health Organization estimates that the global HCC‐related death will reach up to 1 million by 2030 [6]. Therefore, HCC is a serious public health issue worldwide [7].

HCC has an insidious onset, and thus the majority of HCC patients are diagnosed at medium to late stages of the disease [8, 9]. At the advanced stages, treatment options and effectiveness are highly limited, leading to a short survival and high death rate [10]. Therefore, early diagnosis is crucial to reduce the mortality of HCC [4, 11]. To date, serum tumor markers employed in clinics for HCC diagnosis include alpha‐fetoprotein (AFP), alpha‐fetoprotein‐L3 (AFP‐L3), and DCP [12]. In China and Japan, AFP was one of the diagnostic indicators of HCC in the guidelines [13]. However, the sensitivity and specificity of AFP in the diagnosis of HCC are challenged by the fact of underdiagnosis and misdiagnosis [14, 15]. Therefore, AFP is no longer recommended as a diagnostic marker for the screening protocols of HCC in the American Association for the Study of Liver Diseases (AASLD) and the European Association for the Study of the Liver (EASL) [16]. As for AFP‐L3, it is a sub‐type of AFP often tested along with AFP [17]. Clinically, AFP‐L3 may improve the specificity of AFP for HCC diagnosis when tested together, but not the sensitivity [18].

DCP was identified as a protein that could be induced by vitamin K absence, thus also named as PIVKA‐II [19, 20]. Since its identification in 1984 [21], DCP has been recognized as a potential serum marker of HCC, and the diagnostic performance of DCP was reported to be superior to that of AFP [22]. In the literature, however, serum DCP indeed exhibits discrepancies in sensitivity and specificity in the diagnosis of HCC [23]. The combination of DCP and AFP has been shown to have better diagnostic sensitivity than that used alone [24].

Timely assessment of therapeutic efficacy and monitoring of recurrence of HCC are the key to improving the survival and quality of life of patients [4]. To date, AFP is the serum marker utilized most often for monitoring the therapeutic efficacy of HCC [25]. However, the half‐life of serum AFP is 5–6 days, and thus cannot indicate the load changes of HCC in a timely manner [26]. In addition, some HCC patients are negative for AFP or have naturally low AFP levels [27]; in contrast, serum AFP may also be elevated due to non‐HCC conditions. Therefore, there exists a concern regarding AFP as a serum marker for the assessment of therapeutic efficacy [18]. The serum DCP increased in HCC patients has also been used for the assessment of therapeutic efficacy and monitoring of recurrence [28]. The half‐life of DCP is shorter than that of AFP, but the specificity and high baseline level of DCP may be a drawback in clinical practice [26, 29]. It is certainly required for a comprehensive evaluation of clinical applications regarding the specificity and influencing factors of DCP in the diagnosis and therapeutic assessment of HCC. This multicenter large‐scale retrospective study on serum DCP assessed its clinical performance as a diagnostic and therapeutic marker of HCC and identified liver function as a remarkable factor that influences the specificity of serum DCP in the diagnosis of HCC. Serum AFP in the same cohort of patients was evaluated for comparison. The results support DCP as an optimal diagnostic marker of HCC, but attention needs to be paid to the liver function of patients for false positivity; serum DCP may act as a therapeutic marker for surgical and interventional therapies of HCC, but remains elusive in systemic treatment.

## 2 Methods

### 2.1 Ethics

Institutional Review Board (IRB) protocols were approved by the Medical Ethics Committees for Research Involving Human subjects.

### 2.2 Retrospective Data Collections

The retrospective data of 4555 subjects with DCP tests between June 1, 2022 and September 30, 2023, including 311 HCC patients with multiple DCP tests, were collected from Hunan Cancer Hospital, the First Affiliated Hospital of the University of South China, and the Third Hospital of Hebei Medical University. Criteria for inclusion were as follows: naive patients who had serum DCP test results, defined clinical/pathological diagnosis, and complete clinical data. Criteria for exclusion included patients who lacked complete clinical data, had more than one type of tumor diagnosed, or took drugs, such as warfarin and vitamin K. Pregnant or breastfeeding women were also excluded.

The data collected included (1) general demographic characteristics, for example, age and gender; (2) test results of serum tumor markers, for example, DCP, AFP, carcinoembryonic antigen (CEA); (3) medical data, including liver function tests, imaging data (e.g., ultrasound, computed tomography, and MRI), and tumor pathological parameters, such as tumor size, TNM stage, and HCC‐related treatment. According to the discharge diagnosis of patients, the patients involved included PLC (HCC, ICC, HCC‐ICC and histologically unspecified PLC), metastatic liver carcinoma (MLC), benign liver diseases (BLD, e.g., liver cysts, abscesses, liver injury, and hepatic hemangiomas), chronic hepatitis (CH), liver cirrhosis (LC), biliary tract diseases (BTD, i.e., benign diseases of the gallbladder and bile ducts), non‐liver cancers (NLC, e.g., breast cancer, gastric cancer, and abdominal malignant tumors), and non‐liver benign diseases (NLBD, such as gastritis, pancreatic diseases, pneumonia). HCC patients who were continuously monitored for DCP or AFP were grouped for follow‐up analysis.

### 2.3 DCP, AFP, CEA, CA199, and CA125 Data

All data of tumor markers were collected from the hospitals, where DCP was measured with ARCHITECT i2000SR automated immunoassay analyzer and agents from Abbott Laboratories, USA with a Cutoff of 0–40 mAU/mL. AFP was measured with multiple assays including Cobas e601 automated biochemical analyzer and agents from Roche Diagnostics GmbH, Germany with a Cutoff of 0–7 ng/mL, HD‐200A multi‐tumor marker protein detection system and agents from Huzhou Shukang (HealthDigits) Biotechnology Co. Ltd. with a Cutoff of 0–20 ng/mL, ARCHITECT i2000SR automated immunoassay analyzer and agents from Abbott Laboratories, USA with a Cutoff of 0–8.78 ng/mL, and Beckman Coulter, USA with a Cutoff of 0–9 ng/mL. CEA was tested with ARCHITECT i2000SR automated immunoassay analyzer and agents from Abbott Laboratories, USA with a Cutoff of 0–5 ng/mL. CA199 was measured with ARCHITECT i2000SR automated immunoassay analyzer and agents from Abbott Laboratories, USA with a Cutoff of 0–37 U/mL. CA125 was measured with ARCHITECT i2000SR automated immunoassay analyzer and agents from Abbott Laboratories, USA with a Cutoff of 0–35 U/mL.

### 2.4 Statistical Analysis

The data was analyzed using SPSS version 25.0, MedCalc version 15.2.2, and GraphPad Prism version 8.0. Data are presented as mean ± standard error (SE) unless indicated otherwise. As appropriate, ANOVA or the Friedman test was employed to analyze continuous variables. Categorical variables were analyzed using the chi‐square test. SPSS 25.0 was used to perform univariate and multivariate logistic regression analyses. A *p*‐value of < 0.05 was considered statistically significant in all cases.

## 3 Results

### 3.1 Patient Characteristics

This retrospective study cohort included a total of 4555 patients, containing 1814 PLC (39.82%), 218 MLC (4.79%), 580 BLD (12.73%), 226 CH (4.96%), 653 LC (14.34%), 362 BTD (7.95%), 612 NLC (13.44%), and 90 NLBD (1.98%), which were composed of 3018 (66.26%) males and 1537 (33.74%) females. The age of these patients ranged from 34 to 93 years old, with 1902 (41.76%) patients over 55 years old and 2653 (58.24%) patients under 55 years old. Table S1 exhibits the details of subjects involved in this study.

In the 1814 PLC patients, 1041 cases were pathologically diagnosed as HCC, accounting for 57.39%; 166 (9.15%) cases were ICC; and 14 (0.77%) cases were mixed as HCC‐ICC. The rest of 593 (32.69%) cases had not been pathologically subtyped. Table S2 presents the essential data of HCC patients.

### 3.2 Concentrations and Positive Rates of Serum DCP and AFP

**FIGURE 1:**
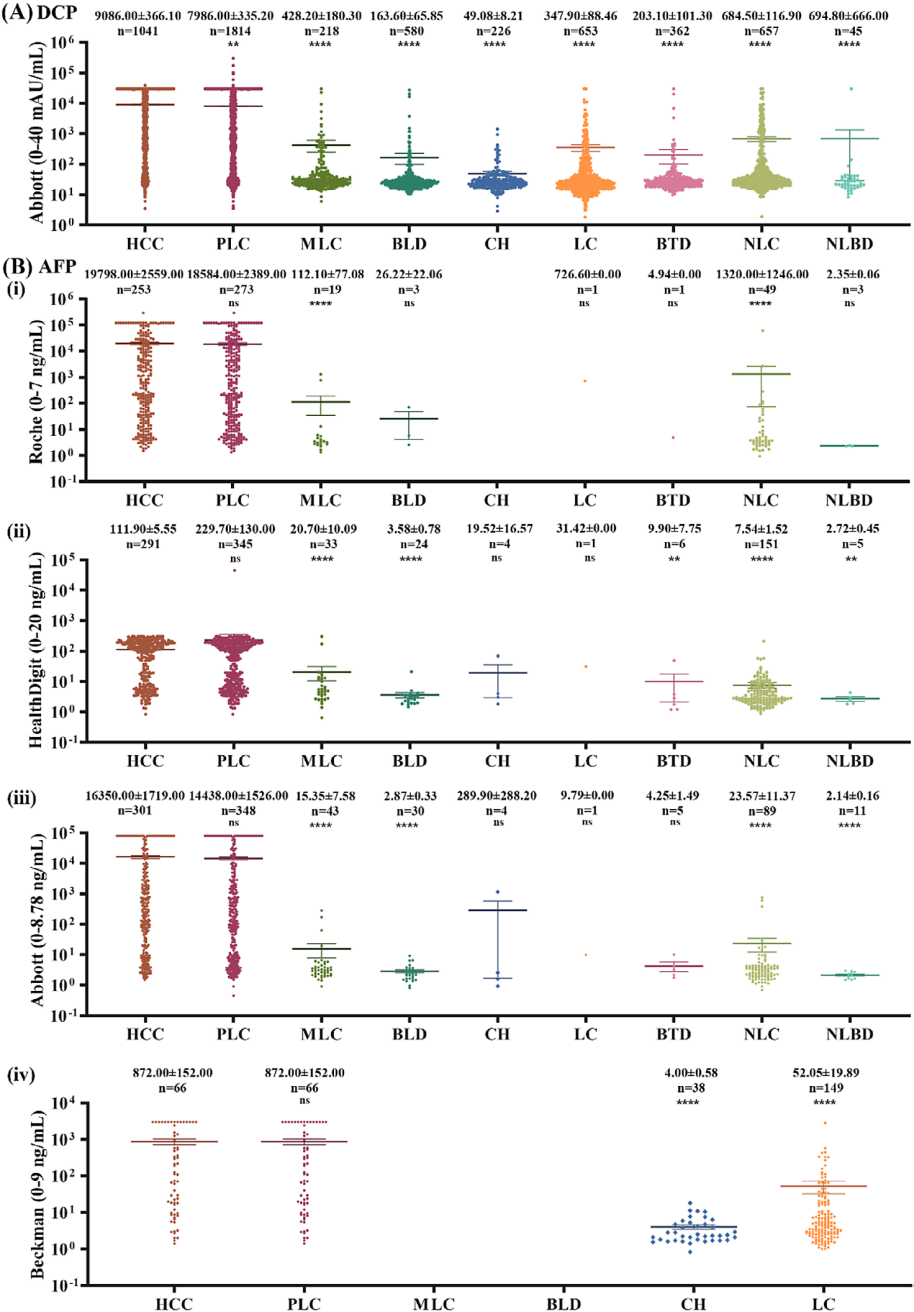
Serum DCP and AFP concentrations in HCC and non‐HCC patients. The serum DCP and AFP data were collected from the study centers and then used for analysis as described in Materials and Methods. (A) Serum DCP concentrations in HCC and non‐HCC patients. (B) Serum AFP concentrations in HCC and non‐HCC patients. Data indicate AFP results tested with different products: Roche (0–7 ng/mL), HealthDigit (0–20 ng/mL), Abbott (0–8.78 ng/mL) and Beckman Coulter (0–9 ng/mL). Data are presented as the mean ± SE. **p < 0.01, ****p < 0.0001, ns, *p* > 0.05, when compared to HCC.

As shown in Figure 1A, serum DCP concentrations in HCC patients were elevated up to 9086.00 ± 366.10 mAU/mL, markedly higher than that in non‐HCC patients (*p* < 0.05). Notably, serum DCP was also increased in some non‐HCC individuals. At the cutoff of 40 mAU/mL suggested by the manufacturer's instruction, positive rates of serum DCP were at 85.11% in HCC, 30.12% in ICC, 31.65% in MLC, 13.95% in BLD, 18.14% in CH, 27.87% in LC, 15.75% in BTD, 35.29% in NLC, and 20.00% in NLBD (Table 1). The positive rate of DCP in HCC was significantly higher than that in non‐HCC diseases, but a substantial proportion of non‐HCC patients appeared positive for serum DCP.

**TABLE 1:** Positive rates of DCP in HCC and non‐HCC patients.

Classification of diseases	DCP		*p*
	Positive (%)	Negative (%)	
PLC	1427 (78.67)	387 (21.33)	
HCC	886 (85.11)	155 (14.89)	
ICC	50 (30.12)	116 (69.88)	
HCC + ICC	11 (78.57)	3 (21.43)	
Undefined	480 (80.94)	113 (19.06)	
MLC	69 (31.65)	149 (68.35)	0.00
BLD	81 (13.95)	499 (86.05)	0.00
CH	41 (18.14)	185 (81.86)	0.00
HBV	24 (13.11)	159 (86.89)	
HCV	2 (22.22)	7 (77.78)	
HBV + HCV	1 (100.00)	0 (0.00)	
HEV	5 (71.43)	2 (28.57)	
ACH	0 (0.00)	2 (100.00)	
Liver Cirrhosis	182 (27.87)	471 (72.13)	0.00
Compensation	3 (33.33)	6 (66.67)	
Decompensation	87 (30.96)	194 (69.04)	
BTD	57 (15.75)	305 (84.25)	0.00
NLC	216 (35.29)	396 (64.71)	0.00
NLBD	18 (20.00)	72 (80.00)	0.00

*Note: p* denotes comparison with HCC.

Serum AFP data were also collected in this retrospective study. Unlike DCP, however, AFP in these study centers was measured with products from different providers, and therefore, the data of serum AFP were grouped and analyzed upon the agents used (Figure 1B). Data showed that serum AFP increased markedly in HCC patients compared to non‐HCC diseases (*p* < 0.05), but it was also noted that serum AFP increased in some non‐HCC patients (Table S3).

In this retrospective study cohort, a portion of patients underwent the tests of serum CEA, CA199, and CA125. As shown in Figure S1 and Table S4, the serum concentrations and positivity of serum CEA, CA125, and CA199 varied with diseases. They are not promising serum markers of HCC.

### 3.3 Diagnostic Performance of Serum DCP and AFP in HCC

Sensitivity and specificity of serum DCP, AFP, CEA, CA199, and CA125 in the diagnosis of HCC were analyzed by chi‐square tests according to the cutoff values recommended by the instructions. As shown in Table S5, serum DCP demonstrated sensitivity and specificity at 85.11% and 75.44%, respectively. Serum AFP tested with the HealthDigit from Huzhou Shukang Biologicals showed specificity and sensitivity similar to that reported in the literature [18], but AFP results measured with other agents showed high variability in sensitivity and specificity. Please see Table S5 for details. The sensitivity and specificity data of CEA, CA199, and CA125 suggested that they were not effective serum markers for HCC diagnosis.

### 3.4 Correlation of Serum DCP With Tumor Pathology and Liver Function

We further assessed the correlation of the serum DCP concentrations with demographic characteristics, tumor pathology, liver function, and other serum tumor markers in HCC patients. As summarized in Table 2, serum DCP in HCC patients positively correlated with gender, tumor size, tumor number, vascular invasion, hepatitis, AFP, ALT, AST, total bilirubin, direct bilirubin, indirect bilirubin, albumin, and A/G ratio (*p* < 0.05), but negatively correlated with age (*p* < 0.05). We also assessed the correlation between the serum DCP and Child‐Pugh grades, but no significant data was observed (Table 2). It is noteworthy that in this retrospective cohort, HCC cases with Child‐Pugh grades were limited (Table S2), which lowered the effective statistical power. A study on a large cohort of HCC cases with Child‐Pugh data is warranted. Serum DCP had no correlation with CEA (*p* = 0.23), CA199 (*p* = 0.39), CA125 (*p* = 0.73), total protein (*p* = 0.05), globulin (*p* = 0.08), and total bile acid (TBA) (*p* = 0.11) (Table 2).

**TABLE 2:** Correlation of serum DCP in HCC patients with clinical parameters and other serum markers.

Parameters	DCP		Parameters	DCP	
	*r*	*p*		*r*	*p*
Gender	0.10	0.00	CA199	−0.03	0.39
Age	−0.55	0.00	CA125	0.01	0.73
Tumor size (cm)	0.48	0.00	ALT	0.18	0.00
Number of tumors	0.13	0.00	AST	0.34	0.00
Portal invasion	0.25	0.00	TBil	0.15	0.00
Liver cirrhosis	−0.06	0.06	DBil	0.14	0.00
Chronic hepatitis	0.07	0.03	IBil	0.18	0.00
AFP (Roche)	0.38	0.00	TP	−0.06	0.05
AFP (HealthDigit)	0.13	0.00	ALB	0.08	0.02
AFP (Abbott)	0.37	0.00	GLB	0.06	0.08
AFP (Beckman Coulter)	0.65	0.00	A/G	0.11	0.00
CEA	−0.27	0.23	TBA	0.05	0.11

### 3.5 Influence of Liver Function on Specificity of Serum DCP in Diagnosis of HCC

In this study cohort, the sensitivity and specificity of serum DCP in the diagnosis of HCC were at 85.11% and 75.44% (Table S5), clearly different from the literature reports [14]. However, this study found that serum DCP was correlated with liver function in addition to tumor pathology (Table 2). Therefore, we further evaluated the diagnostic specificity of serum DCP for HCC stratified by liver function. As shown in Table 3, the diagnostic specificity in HCC of serum DCP was greatly improved when the study cohort was stratified upon various combinations of liver function indicators. When ALT, AST, TBil, DBil, and ALB were combined as a stratification factor, the specificity of DCP in HCC diagnosis reached up to 93.92% with sensitivity at 69.09% in the cohort of normal liver function versus 70.39% with sensitivity at 86.30% in the cohort of abnormal liver function. Similar results were observed in all stratification analyses with different combinations of liver function indices, as well as the Child‐Pugh grades (Table 3). As AFP was measured with different agents with different cut‐off values, the case number for effective statistical power was limited after the stratification, and thus the liver function‐related stratification analysis was omitted.

**TABLE 3:** Diagnostic performance of serum DCP stratified by liver function indicators and tumor pathology.

Parameters		AUC	95% CI	Sensitivity (%)	Specificity (%)	Positive predictive value (%)	Negative predictive value (%)	Accuracy (%)
Liver function								
ALT + AST + TBil + DBil +ALB	Normal	0.82	0.78–0.84	69.09	93.92	50.00	97.19	91.91
ALT + AST + TBil + DBil +ALB	Abnormal	0.78	0.77–0.80	86.30	70.39	56.82	91.92	75.34
ALT + AST + TP + ALB	Normal	0.83	0.80–0.86	73.33	92.98	60.16	96.02	90.49
ALT + AST + TP + ALB	Abnormal	0.78	0.77–0.80	86.77	69.52	56.12	92.13	74.87
ALT + AST	Normal	0.81	0.80–0.83	73.85	88.48	52.94	95.07	86.29
ALT + AST	Abnormal	0.77	0.76–0.79	87.71	67.12	58.06	91.32	74.15
Tumor pathology								
Tumor size (cm)	≥ 5	0.90	0.83–0.94	92.55	87.18	94.57	82.93	90.98
	< 5	0.68	0.60–0.75	73.74	61.77	73.74	61.77	68.86
Number of tumors	Single	0.72	0.66–0.78	79.39	65.15	81.89	61.43	74.62
	Multiple	0.80	0.77–0.84	87.63	73.27	92.64	60.66	84.66
Portal invasion	Yes	0.76	0.74–0.81	90.23	64.81	83.83	76.65	81.82
	No	0.72	0.67–0.78	83.14	61.54	95.50	27.12	81.14

We also conducted stratification analyses on tumor pathology and hepatitis virus infection. In brief, the serum DCP exhibited better diagnostic performance in HCC with advanced or multiple tumors (Table 3). For instance, the diagnostic sensitivity and specificity of serum DCP were 92.55% and 87.18% in HCC of ≥ 5 cm versus 73.74% and 61.77% in HCC of < 5 cm. The serum DCP also showed better diagnostic performance in HCC with portal invasion. We further analyzed the effects of viral infection on the diagnostic performance of DCP. Our data showed that the serum DCP had better diagnostic performance in HCC with HBV infection versus non‐infection, but the HCV infection had not (Table 3).

### 3.6 Serum DCP as a Negative Factor for Prognosis of HCC

Univariate logistic regression was used for risk variable analyses of poor prognosis of HCC patients. As shown in Table 4, factors for poor prognosis of HCC included DCP (OR 15.42, 95% CI 11.73–20.28; *p* < 0.001), CA125 (OR 1.31, 95% CI 1.05–1.65; *p* < 0.05), gender (OR 5.60, 95% CI 4.23–7.42; *p* < 0.001), age (OR 0.54, 95% CI 0.44–0.67; *p* < 0.001), ALT (OR 1.30, 95% CI 1.05–1.60; *p* < 0.05), AST (OR 2.97, 95% CI 2.37–3.73; *p* < 0.001), DBil (OR 2.68, 95% CI 2.12–3.38; *p* < 0.001), IBil (OR 2.19, 95% CI 1.77–2.72; *p* < 0.001), TP (OR 0.77, 95% CI 0.61–0.98; *p* < 0.05), ALB (OR 2.74, 95% CI 2.21–3.39; *p* < 0.001), GLB (OR 1.89, 95% CI 1.37–2.61; *p* < 0.001), and TBA (OR 2.04, 95% CI 1.65–2.51; *p* < 0.001). Collinearity analysis was performed on the statistically significant indicators identified in the univariate logistic regression analysis. After excluding collinearity, a logistic regression analysis with multiple variables was conducted. The results demonstrated that DCP (OR 12.15, 95% CI 8.96–16.48; *p* < 0.001), CA125 (OR 0.74, 95% CI 0.54–0.99; *p* < 0.05), gender (OR 4.17, 95% CI 3.02–5.75; *p* < 0.001), age (OR 0.47, 95% CI 0.36–0.62; *p* < 0.001), ALT (OR 0.43, 95% CI 0.31–0.60; *p* < 0.001), TP (OR 0.35, 95% CI 0.25–0.48; *p* < 0.001), and ALB (OR 2.53, 95% CI 1.85–3.45; *p* < 0.001) were recognized as independent factors for poor prognosis of HCC patients.

**TABLE 4:** Univariate and multivariate logistic regression analysis of serum DCP and clinical pathological parameters in HCC patients.

Parameters	Univariable analysis			Multivariable analysis		
	OR	95% CI	*p*	OR	95% CI	*p*
DCP	15.42	11.73–20.28	0.00	12.15	8.96–16.48	0.00
CEA	0.87	0.66–1.15	0.32			
CA199	0.83	0.67–1.03	0.61			
CA125	1.31	1.05–1.65	0.02	0.74	0.54–0.99	0.04
Gender	5.60	4.23–7.42	0.00	4.17	3.02–5.75	0.00
Age (year)	0.54	0.44–0.67	0.00	0.47	0.36–0.62	0.00
ALT	1.30	1.05–1.60	0.01	0.43	0.31–0.60	0.00
AST	2.97	2.37–3.73	0.00	2.20	1.54–3.14	0.00
TBil	0.95	0.77–1.18	0.64			
DBil	2.68	2.12–3.38	0.00			
IBil	2.19	1.77–2.72	0.00			
TP	0.77	0.61–0.98	0.03	0.35	0.25–0.48	0.00
ALB	2.74	2.21–3.39	0.00	2.53	1.85–3.45	0.00
GLB	1.89	1.37–2.61	0.00			
A/G	1.01	0.82–1.25	0.89			
TBA	2.04	1.65–2.51	0.00			

### 3.7 Serum DCP as a Potential Marker for Surgical Therapy of HCC

**FIGURE 2:**
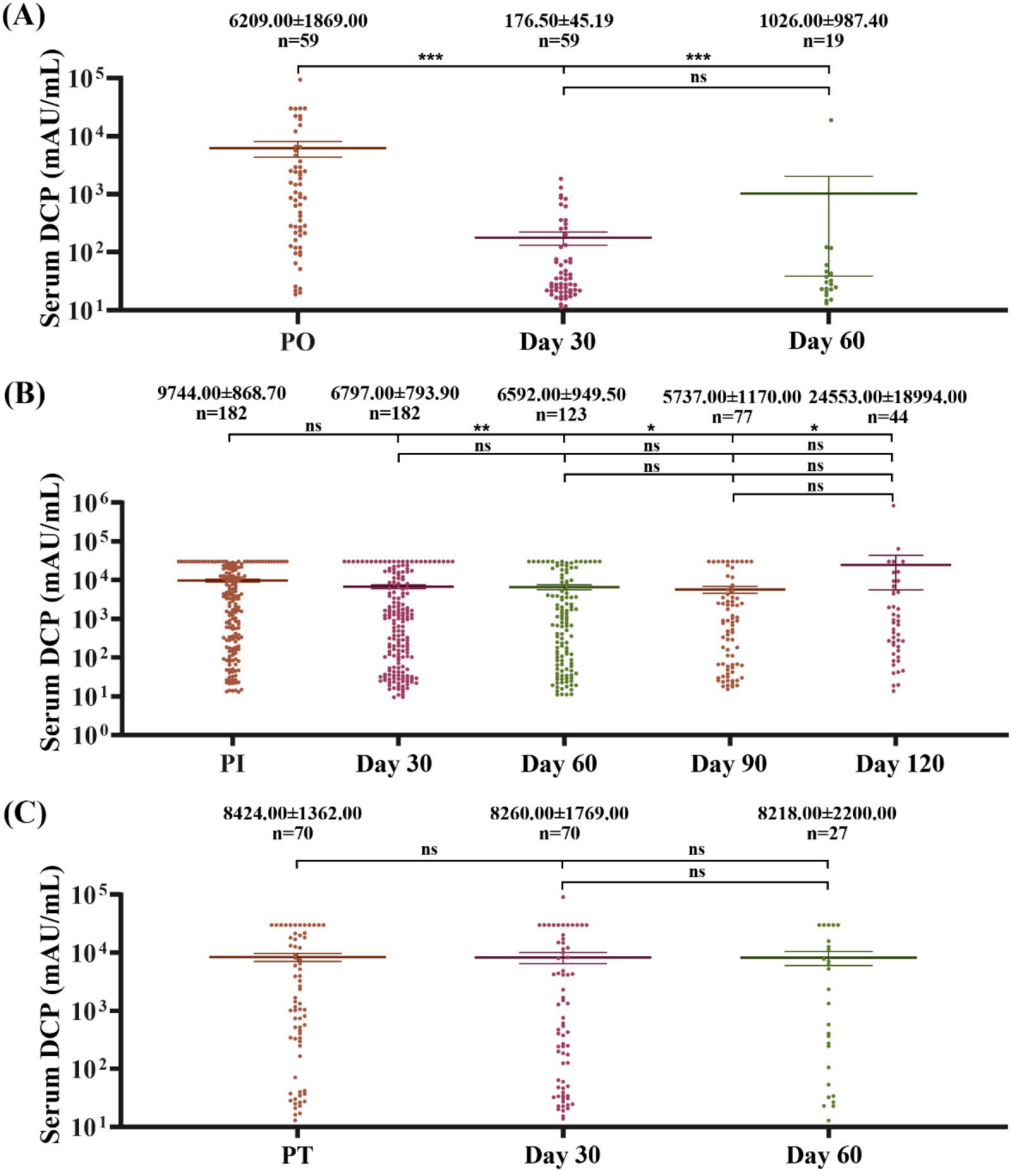
Serum DCP concentrations in HCC patients exposed to various treatments. DCP data for each time point were collected from study centers and then used for analysis as described in Materials and Methods. (A) Serum DCP concentrations in HCC patients with surgical therapy. (B) Serum DCP concentrations in HCC patients receiving interventional therapy. (C) Serum DCP concentrations in HCC patients with drug therapy (TKI plus ICI). *p < 0.05, **p < 0.01, ***p < 0.001, ns, *p* > 0.05, when compared to the serum concentrations before treatment. PO, Pre‐operation; PI, Pre‐interventional; PT, Pre‐drug therapy.

In this retrospective study cohort, 311 HCC cases had multiple DCP tests as they underwent anti‐HCC therapies. In the surgical treatment group, serum DCP in 59 patients was detected on day 30 and day 60 after surgery. The results showed that serum DCP significantly decreased at day 30 after surgery (*p* < 0.0001), but had no notable difference between day 30 and day 60 (*p* > 0.05) (Figure 2A). A total of 182 HCC patients received interventional therapy, in whom serum DCP levels were measured on days 30, 60, 90, and 120 after treatment. The results indicated no significant changes in serum DCP concentrations on day 30 after interventional therapy (*p* = 0.33), which was consistent with the therapeutic responses evaluated via imaging data (Table S6). However, serum DCP declined after the second interventional treatment when compared to the serum DCP levels before the therapy (*p* < 0.05) (Figure 2B). In this cohort, 70 HCC patients received drug treatments, including targeted therapy (TKI) or immunotherapy (ICI), in whom serum DCP was measured on days 30 and 60 after treatment. The results showed no significant changes in serum DCP concentrations in response to treatment (Figure 2C). Table S6 shows the correlation of serum DCP levels to therapeutic responses evaluated via imaging. These data indicate that DCP may serve as a serum marker for HCC resection and interventional therapy, but not optimal for systemic treatments.

In this retrospective study cohort, 256 HCC patients had multiple monitoring of serum AFP levels, and among them, 52 HCC patients had surgical removal, 147 HCC patients underwent interventional therapy, and 57 HCC patients took drug therapy. The serum AFP changes responding to therapeutic responses were similar to those of DCP (Figure S2 and Table S6).

## 4 Discussion

A high rate of HCC incidence and death occurs in China [30]. Early‐stage HCC is asymptomatic, leading to missed optimal treatment options [10], and thus screening and early diagnosis of HCC are critical to improve the survival and quality of life of patients [31, 32, 33]. To date, AFP has been well accepted as a diagnostic marker of HCC due to its convenience, low cost, non‐invasiveness, and repeatability [34, 35]. However, the diagnostic performance of AFP remains suboptimal due to limited sensitivity and specificity [14]. In recent years, serum DCP as a diagnostic marker of HCC has attracted attentions [26, 36], but a large‐scale retrospective evaluation of the clinical performance of DCP is lacking in the Chinese population.

This retrospective study collected the data of 4555 patients and evaluated the diagnostic performance of DCP in HCC. Serum DCP remarkably increased in HCC patients, and at the cutoff value of 40 mAU/mL recommended in the instruction, the positive rate of DCP in HCC was 85.11%, but the specificity was only 75.44% (Table 1). In this cohort of the retrospective study, serum AFP data were produced from multiple agents from different providers, and the positive rates of serum AFP in HCC varied considerably at 84.19% (Roche), 67.35% (Huzhou Shukang), 78.07% (Abbott) and 77.27% (Beckman Coulter), respectively (Table S3). Accordingly, the specificity of serum AFP in the diagnosis of this cohort of HCC varied from 72.73% to 93.28%, indicating the necessity of standardization of AFP tests currently used in clinics.

Serum DCP appeared high false‐positive in this cohort of non‐HCC diseases (Table 1), which may hurt its efficacy in clinical practice. HCC typically develops secondary to hepatitis and liver cirrhosis [37]. This study realized the correlation between serum DCP and liver function (Table 2), which may be attributable to the underlying liver diseases. To evaluate the potential effects of liver function on the clinical performance of DCP, therefore, we conducted a stratification analysis based on various liver function indices, and interestingly, we found that liver function influenced the diagnostic performance of DCP in HCC (Table 3). These findings may partially explain that DCP functioned as an independent risk factor of HCC prognosis (Table 4), being a potential prognostic marker of this disease. In this study, we also found that hepatitis B viral infection influenced the diagnostic performance of serum DCP, but hepatitis C viral infection did not (Table 3). As for tumor pathology, serum DCP demonstrated better diagnostic performance in cases of advanced (> 5.0 cm) or multiple tumors (Table 3). High false‐positive rates of DCP in non‐HCC cases have been reported in the literature, and a clear explanation is still lacking [38, 39, 40, 41, 42]. However, the influence of multiple factors on the sensitivity and specificity of DCP in HCC diagnosis remains unclear, including viral infection, inflammatory lesions, liver reserves, and even the etiology of HCC. Nevertheless, the knowledge of these real clinical phenomena would be instructive in the interpretation of test results.

Data in this study cohort indicated that CEA, CA199, and CA125 were not effective in HCC diagnosis, but interestingly, CA199, an aberrant glycosylation marker [24, 43], had a positive rate of 69.57% in this retrospective cohort of ICC patients, much higher than DCP and AFP (Table S4). CA199 may be a candidate serum marker for ICC diagnosis.

This retrospective study further evaluated the potential of serum DCP and AFP as a therapeutic markers of HCC. Data indicated that the serum DCP concentrations in HCC patients markedly decreased 1 month after surgical removal of HCC, suggesting its capability in the assessment of HCC resection. This study also evaluated the application of serum DCP in interventional and drug treatments of HCC, and the results were effective in patients with more than one interventional therapy, but not promising in drug treatment. It is noteworthy that the assessment was conducted at 1 month after treatment as the half‐life of DCP is 40–72 h [26, 29, 44], emphasizing the need for novel serum markers, such as aldo‐keto reductase 1B10 (AKR1B10) [27], for more timely evaluation and monitoring of treatment response, particularly in systemic treatment. We also conducted this analysis for AFP in HCC patients, but the results are not promising, which may be attributed to the long half‐life of AFP [45]. Further validation is warranted.

Unlike prospective studies, retrospective studies objectively collect and statistically analyze the data from the clinical applications of DCP, which maximally minimizes the influence of subjective factors on the outcomes. However, this retrospective study is limited by an insufficient follow‐up sample size, and the role of DCP in the prognosis of HCC warrants further investigation.

## 5 Conclusion

This multicenter large‐scale retrospective study proves the clinical value of serum DCP in the diagnosis of HCC, particularly in the cases at advanced stages, but its diagnostic performance is markedly influenced by liver function. Attention may need to be paid in the interpretation of DCP tests in clinical practice. Serum DCP may serve as a promising marker for liver resection and interventional therapy in HCC, but its effects in drug treatment remain elusive.

## 6 Recommendations and Future Perspectives

The value of DCP as a therapeutic serum marker needs more extensive evaluation by prospective or retrospective studies. This is particularly important as there is a lack of timely assessment of the therapeutic effects of HCC. The prognostic value of DCP in treatment decision‐making may also be of interest in future clinical observations. Large‐scale, multicentral prospective studies are critical to address this issue. In addition, an emerging strategy in HCC management involves the repurposing of existing drugs and the utility of vitamins, particularly vitamin D, as prophylactic agents with immune and microenvironmental regulatory effects [46]. The effect on serum DCP of these novel agents for HCC is a novel topic to address.

## Author Contributions

Hongying Bu, Meifang Wang, Weijia Luo, and Chen Dong collected the data. Hongying Bu reviewed literature, analyzed the data, drew figures, and wrote the draft. Xu Ye, Xi Zeng, BW, and Qi Yu contributed to the discussion and instructions of contents. Hongyu Deng, Deliang Cao, and Yuemin Nan contributed to study design and revised/finalized the manuscript.

## Conflicts of Interest

The authors declare no conflicts of interest.

## Supporting information


Data S1.

## Data Availability

The data that support the findings of this study are available from the corresponding author upon reasonable request.
