# Supplementary material for: A Large‐Scale Retrospective Study of Serum Des‐Gamma‐Carboxy Prothrombin as a Diagnostic Marker of HCC: Effect of Liver Function on Specificity
Source: J Clin Lab Anal. 2025 Apr 14;39(10):e70025. doi: 10.1002/jcla.70025 (PMC12089794; doi:10.1002/jcla.70025)
Supplement: Supplementary file 1 — Data S1. [file JCLA-39-e70025-s001.docx]

**Table S1. Demographic and clinical data of patients enrolled in this retrospective study (n = 4555)**

| Clinical Parameters | | Number | % | Clinical Parameters | | Number | % |
| --- | --- | --- | --- | --- | --- | --- | --- |
| PLC | | 1814 | 39.82 | TBil (μmol/L) | |  | |
|  | HCC | 1041 | 57.39 |  | Normal | 2438 | 53.52 |
|  | ICC | 166 | 9.15 |  | Abnormal | 1981 | 43.49 |
|  | HCC + ICC | 14 | 0.77 |  | Unmeasured | 136 | 2.99 |
|  | Undefined | 593 | 32.69 | DBil (μmol/L) | |  | |
| MLC | | 218 | 4.79 |  | Normal | 1633 | 35.85 |
| BLD | | 580 | 12.73 |  | Abnormal | 2750 | 60.37 |
| CH | | 226 | 4.96 |  | Unmeasured | 172 | 3.78 |
|  | HBV | 183 | 80.97 | IBil (μmol/L) | |  | |
|  | HCV | 9 | 3.98 |  | Normal | 2865 | 62.9 |
|  | HBV + HCV | 1 | 0.44 |  | Abnormal | 1301 | 28.56 |
|  | HEV | 7 | 3.10 |  | Unmeasured | 389 | 8.54 |
|  | ACH | 2 | 0.88 | TP (g/L) | |  |  |
|  | Undefined | 24 | 10.62 |  | Normal | 2814 | 61.78 |
| Liver Cirrhosis | | 653 | 14.34 |  | Abnormal | 1569 | 34.45 |
|  | compensation | 9 | 1.38 |  | Unmeasured | 172 | 3.78 |
|  | decompensation | 281 | 43.03 | ALB (g/L) | |  |  |
|  | Undefined | 363 | 55.59 |  | Normal | 2370 | 52.03 |
| BTD | | 362 | 7.95 |  | Abnormal | 2049 | 44.98 |
| NLC | | 612 | 13.44 |  | Unmeasured | 136 | 2.99 |
| NLBD | | 90 | 1.98 | GLB (g/L) | |  |  |
| Gender | |  |  |  | Normal | 3747 | 82.26 |
|  | Male | 3018 | 66.26 |  | Abnormal | 419 | 9.2 |
|  | Female | 1537 | 33.74 |  | Unmeasured | 389 | 8.54 |
| Age (y) | |  | | A/G | |  |  |
|  | ≤ 55 | 1902 | 41.76 |  | Normal | 2176 | 47.77 |
|  | > 55 | 2653 | 58.24 |  | Abnormal | 1990 | 43.69 |
| ALT (U/L) | |  | |  | Unmeasured | 389 | 8.54 |
|  | Normal | 2311 | 50.74 | TBA (μmol/L) | |  |  |
|  | Abnormal | 2108 | 46.28 |  | Normal | 2409 | 52.89 |
|  | Unmeasured | 136 | 2.99 |  | Abnormal | 1966 | 43.16 |
| AST (U/L) | |  | |  | Unmeasured | 180 | 3.95 |
|  | Normal | 1758 | 38.59 |  |  |  |  |
|  | Abnormal | 2661 | 58.42 |  |  |  |  |
|  | Unmeasured | 136 | 2.99 |  |  |  |  |

**Table S2. Clinical data of HCC patients (n = 1041)**

| Clinical Parameters | | Number | % | Clinical Parameters | | Number | % |
| --- | --- | --- | --- | --- | --- | --- | --- |
| Gender | |  |  | TBA (μmol/L) | |  |  |
|  | Male | 896 | 86.07 |  | Normal | 422 | 40.54 |
|  | Female | 145 | 13.93 |  | Abnormal | 557 | 53.51 |
| Age | |  |  |  | Unmeasured | 62 | 5.96 |
|  | ≤ 55 | 496 | 47.65 | Hepatitis virus infection | |  |  |
|  | > 55 | 545 | 52.35 |  | Yes | 825 | 79.25 |
| ALT (U/L) | |  | |  | No | 216 | 20.75 |
|  | Normal | 429 | 41.21 |  | Undefined | 0 | 0.00 |
|  | Abnormal | 553 | 53.12 | Liver Cirrhosis | |  |  |
|  | Unmeasured | 59 | 5.67 |  | Yes | 517 | 49.66 |
| AST (U/L) | |  | |  | No | 524 | 50.34 |
|  | Normal | 221 | 21.23 | tumor size (cm) | |  |  |
|  | Abnormal | 761 | 73.10 |  | < 5 | 99 | 9.51 |
|  | Unmeasured | 59 | 5.67 |  | ≥ 5 | 94 | 9.03 |
| TBil (μmol/L) | |  | |  | Undefined | 848 | 81.46 |
|  | Normal | 528 | 50.72 | Portal invasion | |  |  |
|  | Abnormal | 454 | 43.61 |  | Yes | 471 | 45.24 |
|  | Unmeasured | 59 | 5.67 |  | No | 255 | 24.50 |
| DBil (μmol/L) | |  | |  | Undefined | 315 | 30.26 |
|  | Normal | 195 | 18.73 | Number of tumors | |  |  |
|  | Abnormal | 787 | 75.60 |  | single | 131 | 12.58 |
|  | Unmeasured | 59 | 5.67 |  | Multiple | 388 | 37.27 |
| IBil (μmol/L) | |  |  |  | Undefined | 522 | 50.14 |
|  | Normal | 463 | 44.48 | BCLC | |  |  |
|  | Abnormal | 453 | 43.52 |  | 0 | 0 | 0.00 |
|  | Unmeasured | 125 | 12.01 |  | A | 7 | 0.67 |
| TP (g/L) | |  |  |  | B | 29 | 2.79 |
|  | Normal | 680 | 65.32 |  | C | 139 | 13.35 |
|  | Abnormal | 302 | 29.01 |  | Undefined | 866 | 83.19 |
|  | Unmeasured | 59 | 5.67 | TNM | |  |  |
| ALB (g/L) | |  |  |  | Ⅰ | 5 | 0.48 |
|  | Normal | 352 | 33.81 |  | Ⅱ | 4 | 0.38 |
|  | Abnormal | 630 | 60.52 |  | Ⅲ | 7 | 0.67 |
|  | Unmeasured | 59 | 5.67 |  | Ⅳ | 3 | 0.29 |
| GLB (g/L) | |  |  |  | Undefined | 1022 | 98.17 |
|  | Normal | 763 | 73.29 | Child-Pugh classification | |  |  |
|  | Abnormal | 153 | 14.70 |  | A | 4 | 0.38 |
|  | Unmeasured | 125 | 12.01 |  | B | 11 | 1.06 |
| A/G | |  |  |  | C | 1 | 0.10 |
|  | Normal | 473 | 45.44 |  | Undefined | 1025 | 98.46 |
|  | Abnormal | 443 | 42.56 |  |  |  |  |
|  | Unmeasured | 125 | 12.01 |  |  |  |  |

**Table S3. Positive rates of AFP in HCC and non-HCC patients**

| Classification of diseases | | AFP (Roche) | | *p* | AFP (HealthDigit) | | *p* | AFP (Abbott) | |  | AFP (Beckman Coulter) | | *p* |
| --- | --- | --- | --- | --- | --- | --- | --- | --- | --- | --- | --- | --- | --- |
|  |  | Positive (%) | Negative (%) |  | Positive (%) | Negative (%) |  | Positive (%) | Negative (%) |  | Positive (%) | Negative (%) |  |
| PLC | | 224 (82.05) | 49 (17.95) |  | 205 (59.42) | 140 (40.58) |  | 251 (72.13) | 97 (27.87) |  | 51 (77.27) | 15 (22.73) |  |
|  | HCC | 213 (84.19) | 40 (15.81) |  | 196 (67.35) | 95 (32.65) |  | 235 (78.07) | 66 (21.93) |  | 51 (77.27) | 15 (22.73) |  |
|  | ICC | 3 (37.50) | 5 (62.50) |  | 2 (6.90) | 27 (93.1) |  | 7 (23.33) | 23 (76.67) |  |  |  |  |
|  | HCC + ICC |  |  |  | 1 (33.33) | 2 (66.67) |  | 1 (50.00) | 1 (50.00) |  |  |  |  |
|  | Undefined | 8 (66.67) | 4 (33.33) |  | 6 (27.27) | 16 (72.73) |  | 8 (53.33) | 7 (46.67) |  |  |  |  |
| MLC | | 3 (15.79) | 16 (84.21) | 0.00 | 3 (9.09) | 30 (90.91) | 0.00 | 4 (9.30) | 39 (90.70) | 0.00 |  |  |  |
| BLD | | 1 (0.00) | 2 (100.00) | 0.11 | 1 (4.17) | 23 (95.83) | 0.00 | 1 (5.26) | 18 (94.74) | 0.00 |  |  |  |
| CH | |  |  |  | 1 (25.00) | 3 (75.00) | 0.21 | 1 (25.00) | 3 (75.00) | 0.06 | 4 (10.53) | 34 (89.47) | 0.00 |
|  | HBV |  |  |  | 1 (33.33) | 2 (66.67) |  | 1 (25.00) | 3 (75.00) |  | 4 (10.53) | 34 (89.47) |  |
| Liver Cirrhosis | | 1 (100.00) | 0 (0.00) | 1.00 | 1 (100.00) | 0 (0.00) | 1.00 | 1 (100.00) | 0 (0.00) | 1.00 | 47 (31.54) | 102 (68.46) | 0.00 |
| BTD | | 0 (0.00) | 1 (100.00) | 0.36 | 1 (16.67) | 5 (83.33) | 0.03 | 1 (20.00) | 4 (80.00) | 0.01 |  |  |  |
| NLC | | 15 (30.61) | 34 (69.39) | 0.00 | 8 (5.33) | 142 (94.67) | 0.00 | 11 (12.36) | 78 (87.64) | 0.00 |  |  |  |
| NLBD | | 0 (0.00) | 3 (100.00) | 0.00 | 0 (0.00) | 5 (100.00) | 0.01 | 0 (0.00) | 11 (100.00) | 0.00 |  |  |  |

Notes: *p* denotes comparison with HCC.

**Table S4. Positive rates of CEA, CA199, and CA125 in HCC and non-HCC patients**

| Classification of diseases | | CEA | | *p* | CA199 | | *p* | CA125 | | *p* |
| --- | --- | --- | --- | --- | --- | --- | --- | --- | --- | --- |
|  |  | Positive (%) | Negative (%) |  | Positive (%) | Negative (%) |  | Positive (%) | Negative (%) |  |
| PLC | | 180 (18.35) | 801 (81.65) |  | 142 (28.63) | 354 (71.37) |  | 159 (35.73) | 286 (64.27) |  |
|  | HCC | 104 (19.26) | 436 (80.74) |  | 70 (29.17) | 170 (70.83) |  | 28 (35.44) | 51 (64.56) |  |
|  | ICC | 42 (41.18) | 60 (58.82) |  | 32 (69.57) | 14 (30.43) |  | 28 (45.16) | 34 (54.84) |  |
|  | HCC+ICC | 3 (27.27) | 8 (72.73) |  | 2 (40.00) | 3 (60.00) |  | 1 (20.00) | 4 (80.00) |  |
|  | Undefined | 31 (9.45) | 297 (90.55) |  | 38 (18.54) | 167 (81.46) |  | 102 (34.11) | 197 (65.89) |  |
| MLC | | 83 (55.33) | 67 (44.67) | 0.00 | 58 (69.88) | 25 (30.12) | 0.00 | 45 (46.88) | 51 (53.12) | 0.13 |
| BLD | | 7 (2.76) | 247 (97.24) | 0.00 | 22 (19.64) | 90 (80.36) | 0.06 | 29 (10.36) | 251 (59.64) | 0.00 |
| CH | | 9 (12.50) | 63 (87.50) | 0.17 | 13 (19.40) | 54 (80.60) | 0.11 | 15 (23.81) | 48 (76.19) | 0.13 |
|  | HBV | 8 (14.04) | 49 (85.96) |  | 11 (26.19) | 31 (73.81) |  | 14 (25.00) | 42 (75.00) |  |
|  | HCV | 1 (25.00) | 3 (75.00) |  | 1 (16.67) | 5 (83.33) |  | 1 (33.33) | 2 (66.67) |  |
|  | HEV | 0 (0.00) | 3 (100.00) |  | 0 (0.00) | 3 (100.00) |  | 0 (0.00) | 2 (100.00) |  |
| Liver Cirrhosis | | 32 (17.20) | 154 (82.80) | 0.54 | 32 (14.29) | 192 (85.71) | 0.00 | 78 (44.57) | 97 (55.43) | 0.17 |
|  | compensation | 0 (0.00) | 2 (100.00) |  |  |  |  | 0 (0.00) | 1 (100.00) |  |
|  | decompensation | 18 (18.75) | 78 (81.25) |  | 18 (12.50) | 126 (87.50) |  | 53 (80.30) | 13 (19.70) |  |
| BTD | | 2 (1.39) | 142 (98.61) | 0.00 | 22 (31.43) | 48 (68.57) | 0.72 | 19 (9.79) | 175 (90.21) | 0.00 |
| NLC | | 140 (31.82) | 300 (68.18) | 0.00 | 121 (59.02) | 84 (40.98) | 0.00 | 46 (24.47) | 142 (75.53) | 0.07 |
| NLBD | | 6 (13.64) | 38 (86.36) | 0.36 | 4 (14.29) | 24 (85.71) | 0.10 | 9 (21.95) | 32 (78.05) | 0.13 |

Notes: *p* denotes comparison with HCC.

**Table S5. Diagnostic performance of serum DCP, AFP, CEA, CA199, CA125 in HCC**

| Parameters | Number | AUC | 95% CI | Sensitivity (%) | Specificity (%) | Positive predictive value (%) | Negative predictive value (%) | Accuracy (%) |
| --- | --- | --- | --- | --- | --- | --- | --- | --- |
| DCP (Abbott) | 4555 | 0.80 | 0.79 - 0.82 | 85.11 | 75.44 | 55.38 | 93.40 | 77.99 |
| AFP (Roche) | 349 | 0.79 | 0.74 – 0.83 | 84.19 | 73.33 | 91.42 | 57.90 | 81.71 |
| AFP (HealthDigit) | 568 | 0.80 | 0.77 – 0.84 | 67.35 | 93.28 | 92.02 | 71.30 | 79.41 |
| AFP (Abbott) | 520 | 0.82 | 0.79 - 0.86 | 78.07 | 86.63 | 90.39 | 71.05 | 81.35 |
| AFP  (Beckman Coulter) | 523 | 0.75 | 0.69 - 0.80 | 77.27 | 72.73 | 50.00 | 90.07 | 73.91 |
| CEA (Abbott) | 2271 | 0.48 | 0.46 - 0.50 | 19.26 | 76.94 | 24.47 | 71.07 | 60.82 |
| CA199 (Abbott) | 1285 | 0.47 | 0.44 - 0.50 | 29.17 | 64.02 | 18.72 | 76.09 | 56.31 |
| CA125 (Abbott) | 1482 | 0.53 | 0.50 - 0.55 | 31.80 | 73.17 | 27.60 | 76.93 | 63.10 |

**Table S6. Association of DCP and AFP with the Efficacy of Interventional and Pharmacological Treatments**

| Parameters | | DCP | | | |  | AFP | | | |
| --- | --- | --- | --- | --- | --- | --- | --- | --- | --- | --- |
|  |  | Increase (number) | Decrease  (number) | r | p |  | Increase  (number) | Decrease  (number) | r | p |
| interventional therapy | Effective | 37 | 86 | -0.15 | 0.06 |  | 40 | 83 | -0.12 | 0.15 |
|  | Ineffective | 17 | 14 |  |  |  | 16 | 15 |  |  |
|  |  |  |  |  |  |  |  |  |  |  |
| drug therapy (TKI plus ICI | Effective | 11 | 14 | -0.23 | 0.18 |  | 7 | 18 | -0.39 | 0.02 |
|  | Ineffective | 8 | 3 |  |  |  | 6 | 5 |  |  |


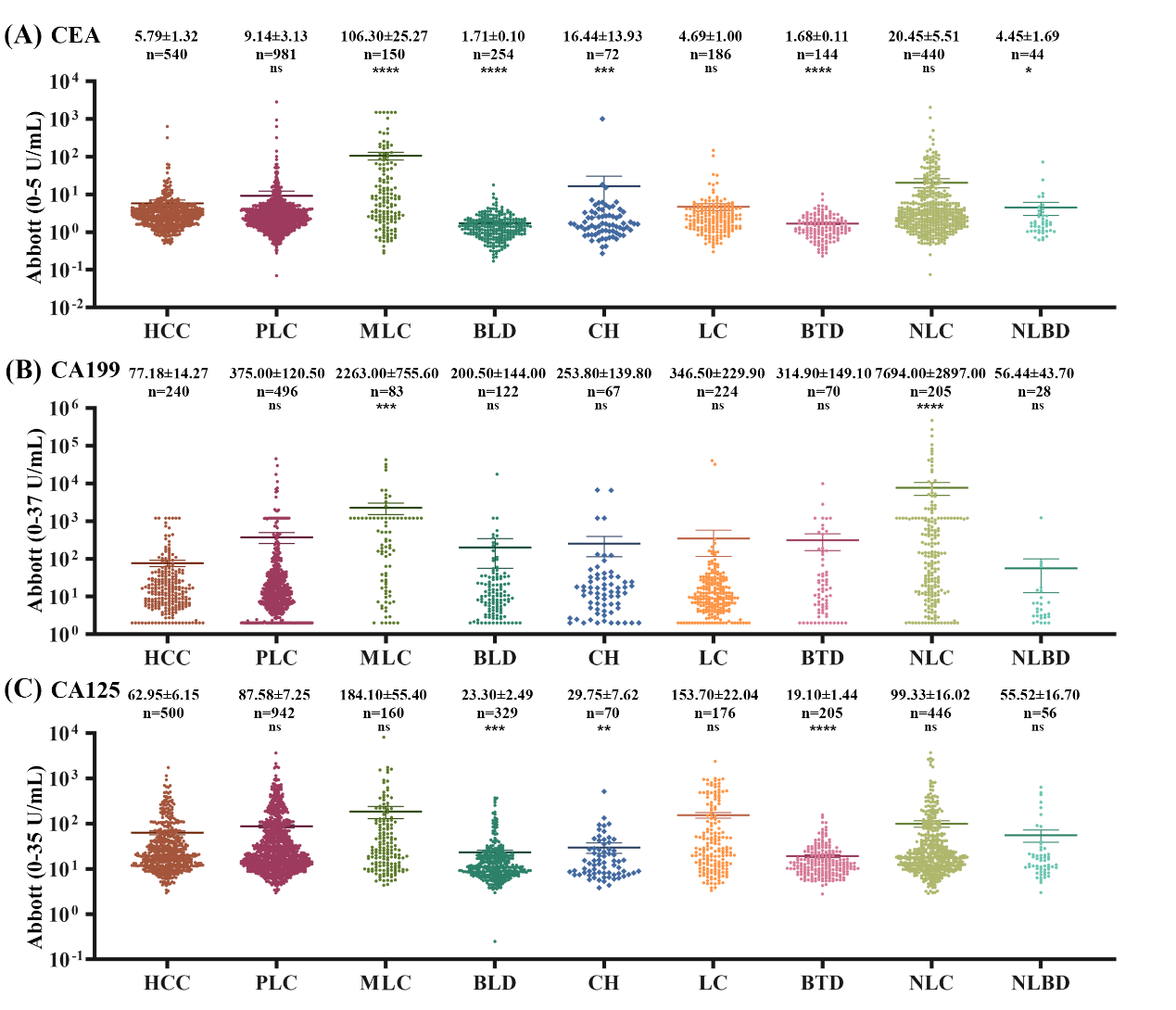


**Fig S1. Serum** **CEA, CA199 and CA125 concentrations in HCC and non-HCC patients.** The serum CEA, CA199 and CA125 data were collected from the study centers and then used for analysis as described in Materials and Methods. (A) Serum CEA concentrations in HCC and non-HCC patients. (B) Serum CA199 concentrations in HCC and non-HCC patients. (C) Serum CA125 concentrations in HCC and non-HCC patients. Data are presented as the mean ± SE. **p* < 0.05, ***p* < 0.01, ****p* < 0.001, *****p* < 0.0001, ns, *p* > 0.05, when compared with HCC.


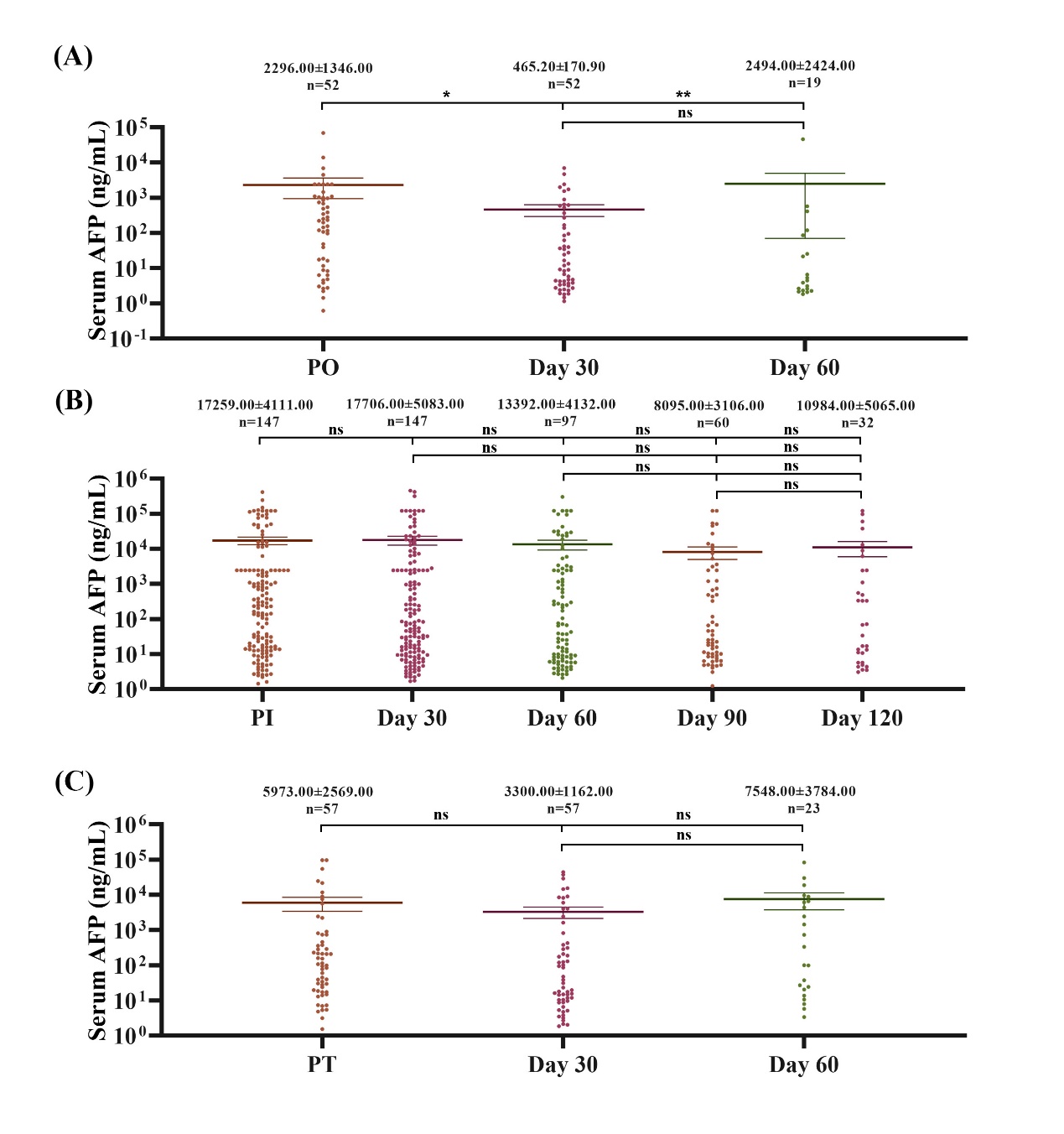


**Fig S2. Serum AFP concentrations in HCC patients exposed to various treatments.**  AFP data for each time point were collected from study centers and then used for analysis as described in Materials and Methods. (A) Serum AFP concentrations in HCC patients with surgical therapy. (B) Serum AFP concentrations in HCC patients receiving interventional therapy. (C) Serum AFP concentrations in HCC patients with drug therapy (TKI plus ICI). *p < 0.05, **p < 0.01, ***p < 0.001, ****p < 0.0001, ns, p > 0.05, when compared to the serum concentrations before treatment. PO, Pre-operation; PI, Pre-interventional; PT, Pre-drug therapy.
